# Evaluation of Prefrontal Cortex Activation and Static Balance Mechanisms in Adolescent Idiopathic Scoliosis Using fNIRS

**DOI:** 10.3390/medicina61040667

**Published:** 2025-04-04

**Authors:** Esra Suzen, Kadriye Tombak, Buket Simsek, Omer Halil Colak, Sukru Ozen

**Affiliations:** 1 NeuroscienceLab, Faculty of Engineering, Department of Electrical and Electronics Engineering, Akdeniz University, Antalya 07058, Turkey; simsekbukett@gmail.com (B.S.); omercol@akdeniz.edu.tr (O.H.C.); sukruozen@akdeniz.edu.tr (S.O.); 2 Department of Physical Therapy, Vocational School of Health Services, Akdeniz University, Antalya 07058, Turkey

**Keywords:** adolescent idiopathic scoliosis, functional near-infrared spectroscopy, postural control, brain activation, prefrontal cortex activity, oxyhemoglobin

## Abstract

*Background and Objectives:* In this study, the role of oxyhemoglobin (HbO) in subregions of the prefrontal cortex during a static balance task under eyes-open and eyes-closed conditions was investigated in adolescent idiopathic scoliosis (AIS) using functional near-infrared spectroscopy (fNIRS), a powerful neuroimaging tool that enables more natural and flexible measurement in the analysis of balance mechanisms and motor tasks. *Materials and Methods:* Hemodynamic changes in the right and left dorsolateral cortex (DLPFC), frontopolar prefrontal cortex, and orbitofrontal cortex were analyzed in 16 healthy controls and 15 individuals with AIS. The statistical results were supported by HbO contrast maps. *Results:* Significant differences were found in the cortical activity patterns between the AIS and control groups. The AIS group had lower HbO concentrations than the control group in the eyes-closed condition and completely differed from the control group by showing more active HbO concentrations in the DLPFC regions than in the frontopolar regions. In the eyes-open condition, it was found that the maximum HbO value was reached in the frontopolar regions, and this value was weakened and observed throughout the left frontopolar region. Discriminative differences were also found in the orbitofrontal region in the eyes-closed static balance condition. *Conclusions:* The results obtained were evaluated and discussed in terms of postural balance compensation, differences in neural pathways, and the conscious balance mechanism. It was determined that the AIS group tended to utilize a conscious balance mechanism in the eyes-closed static balance condition and developed its own balance compensation mechanism in the eyes-open static balance condition. This study concludes that fNIRS is a powerful tool in the evaluation of balance and control mechanisms and can be used effectively in the evaluation of rehabilitation-oriented development in AIS.

## 1. Introduction

Scoliosis significantly affects quality of life, as it changes individuals’ perceptions of health and can potentially lead to disability [1]. Scoliosis is a complex, three-dimensional structural deformity of the spine that involves a lateral deviation of more than 10° in the frontal plane, axial (vertebral) rotation in the horizontal plane, and changes in the physiological limits of flexion and extension (kyphosis and lordosis) in the sagittal plane [2]. The calculation of the increase in the Cobb angle and the determination of risk factors are performed within the framework of published guidelines [3]. Although there are many subtypes of scoliosis, the most common type is adolescent idiopathic scoliosis (AIS), which is reported to affect approximately 0.47% to 5.2% of adolescents worldwide. In individuals with AIS, the most important goal is to develop the correct posture and increase their body awareness in their daily activities through exercise [4,5].

Exercise treatment is frequently recommended for individuals with scoliosis classified as mild to moderate, with curvatures ranging from 10° to 30°. If the desired awareness is not achieved through exercise, several factors, including asymmetric loading and muscle imbalances, can negatively affect the development of the spine. As a result, the rotational components of the curves of these individuals increase, and secondary complications occur during the transition from adolescence to adulthood and beyond [6,7,8,9]. These secondary problems may include pain, muscle imbalances, functional deficits, cosmetic problems, distraction, and focusing difficulties.

Understanding the neurocognitive basis of balance processes in adolescents with adolescent idiopathic scoliosis (AIS) is critical because brain oscillations, through neural plasticity, can be reorganized in the system in an attempt to compensate for the dysfunction [10].

Rehabilitation efforts are also of great importance in managing these processes. The apparent ease of standing in the healthy individual conceals the underlying neuromotor processes. The basal ganglia and cerebellum may influence both automatic and cognitive postural control processes through reciprocal connections with the brainstem and cerebral cortex, respectively [11]. Thus, in addition to damage to the motor cortex, basal ganglia, and cerebellum, impairments in cognitive function can impair postural control and lead to falls [12,13]. The position of the human body is perceived via sensory inputs by the somatosensory, visual, and vestibular systems that are sent to the central nervous system, consisting of the brain and spinal cord [14,15]. The cooperation of the vestibulospinal, reticulospinal, and tectospinal tracts contributes significantly to this process. Sensory information is integrated at this level, resulting in the generation of motor commands directed toward the musculoskeletal system to initiate muscle contractions and adjust the body position [14]. Accordingly, various regions of the cerebral cortex, particularly the frontal areas, play a critical role in maintaining postural stability [16]. On the other hand, tasks such as walking under unusual or challenging conditions require not only reflexive responses but also the involvement of higher-order cognitive processes. These processes rely on body schema, the perception of postural verticality, and the internal representation of the body’s motion in space, collectively referred to as bodily self-perception [13]. The limited investigation of specific structures within the frontal cortex under postural control conditions in the current literature continues to pose a barrier to a full understanding of this complex mechanism [11].

Electroencephalography (EEG) studies show that AIS adolescents have increased cortical activation in the frontal, parietal, and occipital regions to control balance during standing postures [17,18]. In addition, the theta activity has been reported to increase in the parietal and frontal areas with an increase in the balance demand. Although it has been suggested that this increase may support postural control during balance tasks, some studies have found that the difference between individuals with AIS and healthy individuals is not significant [18,19,20]. MRI studies report differences in white matter structures, the vestibular system, and motor-related areas in individuals with AIS [21]. According to a review of studies published in the last 15 years, neurological changes in individuals with AIS have been examined with methods such as EEG, electromyography (EMG), functional magnetic resonance imaging (fMRI), somatosensory evoked potentials, force platforms, and motion capture, and the increasing interest in neurophysiological assessments has been emphasized [21]. Although the available evidence suggests that individuals with AIS have impairments in standing balance and postural control, it is noteworthy that the functional near-infrared spectroscopy (fNIRS) method, which has become widespread in recent years, has not yet been used in this field for AIS. fNIRS is a technique that indirectly assesses brain activity by measuring the hemodynamic responses of cortical tissues. This method sends near-infrared light through the scalp and skull, analyzing the intensity changes of the light reflected and diffusely refracted from the tissues. Neural activation in response to a stimulus causes an increase in blood flow in the affected area, which leads to local changes in the concentrations of oxygenated hemoglobin (HbO), deoxygenated hemoglobin (HbR), and total hemoglobin (HbT) [22]. Portable fNIRS devices allow for the investigation of neural correlates of moving tasks such as walking, which is not possible with fMRI [23,24]. Despite its lower resolution compared with fMRI, fNIRS offers a wider range of applications due to its portability and ease of applicability [25]. Performing fMRI measurements in a limited area where the patient’s movement is not possible limits the evaluation of the change process created by motor movements in the cortex to EEG-solution-focused results. One of the most important advantages of fNIRS as a brain imaging tool is that it has no strict restrictions on movement. This increases the range of possibilities for researchers and clinicians by ensuring high data quality and eliminating motion artifacts, eliminating the need to be stationary during acquisition. These properties of fNIRS make it an ideal tool for the study of balance and motor control processes [11]. fNIRS research into balance performance is at a relatively early stage and needs to be expanded in different directions. The review study by Baradaran et al. supports the use of fNIRS measurement in combination with quantitative balance assessment to provide a better understanding of balance neuromotor control in different populations [26].

The frontal cortex is the area responsible for executive functions necessary for balance control, voluntary movement, and cognitive behavior. Therefore, the frontal cortex and its subregions are the most frequently examined regions in balance function [26]. In our study, we performed a detailed analysis of the prefrontal area during static balance in AIS and quantitatively investigated the differences with healthy adolescents. This is the first fNIRS-based study that fills the gap in the literature in the field of postural control neuroimaging and the analysis of static balance in individuals with AIS.

## 2. Materials and Methods

This cross-sectional study was conducted in healthy adolescents and adolescents diagnosed with scoliosis by relevant physicians. The study received ethical approval from the Akdeniz University Faculty of Medicine Clinical Research Ethics Committee (KAEK-179, 22 February 2023). Since all participants were under the age of 18 years, written and verbal consent to participate in the study was obtained from the parents of the participants. The study was conducted in accordance with the Declaration of Helsinki.

### 2.1. Participants

The inclusion criteria in the AIS group were right-handed dominant use, being diagnosed with AIS by a relevant physician, being between the ages of 8 and 18 years, and having a Cobb angle between 10 and 35 degrees. Exclusion criteria were the presence of scoliosis other than AIS; neurologic, psychiatric, muscular, rheumatic, or orthopedic diseases; and having undergone scoliosis surgery. A total of 31 volunteers, including 16 healthy subjects (12.75 ± 2.64) and 15 AIS subjects (14.47 ± 2.615) with the demographic characteristics given in Table 1, were included in our study. Since fNIRS was not used in the field of neuroimaging and analysis in the AIS population in the literature review, data such as means and standard deviations could not be obtained, so the sample size was not calculated.

### 2.2. Experimental Setup and Study Design

In this study, the participants were asked to stand with their feet shoulder-width apart and hands straight down at both sides, free and motionless. During this posture, fNIRS recordings were taken for 30 s each in the eyes-open (EO) and eyes-closed (EC) conditions by looking at a fixed point at eye level and 2 m away from the participant. A rest period of 5 min was given between EO and EC recordings. If the static balance position was disturbed, the recording was canceled and repeated.

### 2.3. fNIRS Data Acquisition

Functional near-infrared spectroscopy (fNIRS) was used to determine prefrontal cortex (PFC) activity in the AIS and control groups. Recordings were performed with a portable continuous-wave NIRSport system (NIRSport2© NIRx Medical Technologies, Berlin, Germany) with two wavelengths (760 nm, 850 nm). Data were acquired with the Aurora data acquisition software (v2021.4 NIRx Medical Technologies LLC, Berlin, Germany) with a 4 Hz low-pass filter, 3.0 µV < dark noise < 15.0 µV, and a sampling frequency of 10.2 Hz.

**Figure 1:**
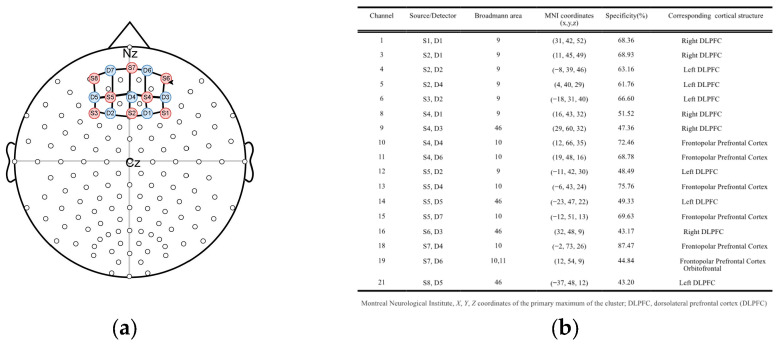
fNIRS optode placement and areas of interest: (**a**) fNIRS configuration covering the prefrontal cortex (red circles are source channels, blue circles are detector channels, and their interconnections are shown); (**b**) channels represented by the channel/detector pairs used, their corresponding Brodmann regions, the MNI coordinates, the specificity percentages, and the corresponding cortical structures.

According to the international 10/20 system [27], the optodes were fixed to the prefrontal cortex region of the participant (8 LED sources and 7 detectors) using a headband. The source–detector distance was 3 cm. The 22-channel optode placement was evaluated according to Brodmann areas (BAs) using the fNIRS Optodes Location Decider (fOLD) MATLAB 2017b toolbox [28]. The optode configuration covered BA9, BA10, BA11, and BA46 with 37% specificity. This configuration allowed the assessment of the activation of brain regions associated with concentration, attentional control, cognitive control, complex planning, and focusing. Figure 1 shows the MNI coordinates and brain areas of the channels.

### 2.4. fNIRS Data Processing and Analyses

The raw fNIRS data were transferred to MATLAB (R2017b, The MathWorks Inc., Natick, MA, USA). The fNIRS processing package, namely the HOMER3 MATLAB toolbox [29], was used to isolate cortical activity from motion artifacts and systemic noise and to convert it into concentration changes. The raw intensity data were first converted into optical density data. Subsequently, motion artifacts, physiological noise, and instrumentation-related noise were removed using filtering and correction algorithms. Physiological noise arises from the heartbeat, respiration, and blood pressure fluctuations. High-frequency oscillations correspond to cardiac activity (1~1.5 Hz), whereas low-frequency oscillations correspond to Mayer waves [30]. To eliminate such physiological noise, a band-pass filter between 0.01 and 0.20 Hz was applied [31,32]. Experimental errors, such as head movements causing the optodes to shift from their designated positions, lead to sudden changes in light intensity. Instrumental noise, often containing constant high frequencies, results from the device hardware or environmental factors. At this stage, the hmrMotionArtifactbyChannel function in HOMER3 was used to detect motion artifacts (SDthresh = 50, AMPthresh = 5, tMask = 1 s) [29].

Then, to correct the artifacts in the optical density signals, wavelet-based and hybrid spline interpolation–Savitzky–Golay filtering methods were applied. Wavelet filtering is effective in eliminating abrupt motion-induced signal changes [33], while spline interpolation corrects baseline shifts, and high-frequency artifacts are attenuated using Savitzky–Golay (SG) filtering [34]. Wavelet-based filtering was applied using the hmrMotionCorrectWavelet function in HOMER3 [29]. The wavelet transform provides an advantage in distinguishing motion artifacts by optimizing the time–frequency resolution. Coefficients below the threshold determined using a Gaussian model were preserved, while those above the threshold were set to zero as artifacts. The cleaned fNIRS signal was obtained through an inverse wavelet transform. For computational efficiency, the Daubechies 2 (db2) wavelet was used, and outliers were identified using the interquartile range (IQR = 1.5) method [33].

The hybrid spline interpolation–Savitzky–Golay filtering was implemented using the MotionCorrectSplineSG function in HOMER3 (*p* = 0.99, FrameSize = 10 s) [29,34]. To correct baseline shifts, a motion artifact modeling method was applied using cubic spline interpolation, while sudden spikes were corrected using the Savitzky–Golay filter. This filtering method reduces noise while preserving the signal characteristics by fitting a polynomial to the specified data window.

The cleaned data were then converted into HbO and HbR concentration changes using the modified Beer–Lambert law (MBLL) [35,36]. Finally, block averaging was performed using the hmrBlockAvg function (trange = [−2.0, 20.0]). The relative HbO and HbR concentrations reflect neural activity [37]. In this study, comparisons were performed based on the HbO values, as HbO responses are more commonly observed than HbR [38,39,40], show lower inter-individual variability [41,42,43], and have a higher signal-to-noise ratio, while being less affected by factors such as cross-talk [42,44,45]. The projection and visualization of the HbO values on the cortical surface were carried out using the AtlasViewer MATLAB toolbox, which employs the “Colin27” digital brain atlas [46,47].

### 2.5. Statistical Analysis

The SPSS version 27.0 (IBM Inc., Armonk, NY, USA) program was used for the statistical analysis of the findings obtained in this study. The suitability of continuous variables in terms of a normal distribution was examined with the Kolmogorov–Smirnov test. In order to describe the sample, variables with a normal distribution were expressed as the mean ± standard deviation, variables without a normal distribution were expressed as the median (minimum–maximum), and categorical variables were expressed as a number and percentage. The chi-squared test of independence was used to analyze gender differences. Since the BMI variable did not show a normal distribution, the Mann–Whitney U test was used to compare the groups. The independent-sample *t*-test was used to determine the differences between the AIS and control groups in the HbO concentration in 22 channels in the eyes-open and eyes-closed static balance conditions. In the analyses, a 95% significance level (or α = 0.05 margin of error) was used to determine differences.

## 3. Results

The differences obtained between the two groups in the HbO concentration in the static balance condition in 22 channels with eyes open (EO) and eyes closed (EC) are presented in Table 2 and Table 3. A statistically significant difference (*p* < 0.05) was observed in channels 12, 13, and 18 in the left DLPFC and frontopolar prefrontal cortex areas in the comparison between the AIS and control groups in static balance with eyes open. In the other channels, the AIS group showed similar HbO concentration values to the control group (Table 2).

A statistically significant difference (*p* < 0.05) was observed between the AIS and control groups in channels 14, 15, 16, 18, and 20 in the right–left dlPFC, frontopolar prefrontal cortex, and orbitofrontal cortex areas in static balance with eyes closed. In the other channels, the AIS group showed similar HbO concentration values to the control group (Table 3).

Table 4 shows the topographic maps of the control and AIS groups under the eyes-open and eyes-closed static balance conditions. The increased difficulty of the eyes-closed balance condition compared to the eyes-open condition led to more diffuse activation patterns in the topographic maps of both groups. While a more localized activation was observed in the control group under the eyes-open static balance condition, activation in the AIS group extended into the left frontopolar region, albeit attenuated. This may indicate that the control group was able to maintain balance under the eyes-open condition with reduced cortical resource utilization. In the AIS group, peak activation was also observed in the left frontopolar region, which may suggest that individuals with AIS require a larger cortical area to maintain balance.

Under the eyes-closed static balance condition, the AIS group exhibited a symmetrical activation pattern in the left and right DLPFC regions, demonstrating a distinctly different activation profile compared to the control group. In contrast, the control group showed a weakening activity pattern beginning in the left-dominant frontopolar region and extending toward the DLPFC. In the eyes-closed condition, the AIS group displayed significantly less activity in the frontopolar region compared to the control group, indicating differences in postural control strategies. Significant differences were also observed in the orbitofrontal region. The differences in the HbO values in the orbitofrontal region—markedly weak in the AIS group but active in the control group under the eyes-closed (EC) condition—were statistically confirmed and reflected in the contrast map. These findings suggest that individuals with AIS employ distinct neural mechanisms compared to the healthy population in order to maintain postural stability.

## 4. Discussion

Analyzing the prefrontal cortex using fNIRS is crucial in understanding the underlying mechanisms of balance and motor control processes in the AIS population. This analysis provides a better understanding of the functioning of the prefrontal cortex and its sub-areas during balance control and the compensatory strategies used by the AIS population to maintain balance.

Analyses of the prefrontal cortex sub-areas revealed that the DLPFC was frequently selected due to its well-known connection with executive function, attention, and balance control [48,49]. The DLPFC is involved in providing attentional resources for the maintenance of postural control and the integration of external information with information about the body position [50]. In the fMRI study conducted by Park et al., it was reported that the DLPFC is more active during the early stages of motor learning and plays a role in the allocation of attentional resources, as well as the integration of external information with body position awareness [51]. When examining fNIRS studies in the literature, Mihara and colleagues demonstrated that, after postural disturbances, there was a significant increase in HbO activation in the bilateral DLPFC [52]. Similarly, another study conducted by Mihara and colleagues with elderly hemiplegic stroke patients showed significant increases in DLPFC HbO signals following anterior–posterior and mediolateral postural deviations [53]. Similarly, in our study, it was observed that the AIS group exhibited higher DLPFC activation than the control group during EO static postures (Table 2). However, when the AIS group was analyzed separately, the frontopolar region demonstrated stronger HbO activity than the DLPFC, as illustrated in the contrast map (Table 4). This finding suggests that the AIS population may exhibit distinct activation patterns in regions associated with motor control and attention. In the control group, the peak activation and primary activity region were located in the left DLPFC, aligning with the literature that highlights the DLPFC’s role in postural control and executive function. Notably, the difference in left DLPFC activity between the AIS and control groups during EO static posture supports its contribution to balance control.

The alignment of the hemodynamic response differences observed in our study with the findings from fMRI [51] and EEG studies [54] on postural control and balance further underscores the critical role of the DLPFC and demonstrates the effectiveness of fNIRS in identifying these mechanisms. The increased left DLPFC activation in the AIS group may indicate that these individuals exert greater cognitive effort during balance control or develop an alternative connectivity mechanism. This finding has significant implications for future research and strengthens the contribution of our study to the field.

In the absence of visual feedback, proprioceptive and vestibular information becomes more important in maintaining balance. In our study, lower HbO values were observed in the AIS group during eyes-closed (EC) static balance compared with the control group. Supporting this finding, a recent study investigated neural activity in the prefrontal cortex (PFC) using fNIRS in individuals with chronic neck pain who exhibited impaired postural control. It was observed that, during eyes-closed static standing, both the left and right PFC showed lower activation compared with healthy controls [55]. The higher HbO values observed in the control group indicate that these individuals utilize sensory feedback more effectively and, consequently, are better able to maintain balance. The orbitofrontal cortex is a sub-area of the prefrontal cortex that is involved in decision-making, sensory integration, and reward–motivation mechanisms, as well as postural control and the processing of environmental feedback. The low HbO values in the orbitofrontal cortex in the AIS group suggest a deficit in sensory integration. This situation affects postural control. The frontopolar cortex, one of the subregions of the prefrontal cortex, presents differences in static balance. The frontopolar cortex occupies the anterior part of the frontal lobe of the brain and plays a role in imagination [56]. Accordingly, after the eyes are closed, mental activity shifts from an external to an internal state, characterized by imagination and multisensory activity, depending on information from the frontopolar cortices [57].

Helmich et al. conducted a fNIRS study on concussed athletes in a static balance state with eyes closed and found a decrease in oxygenation in the frontopolar cortex area [58]. Similarly, in our study, lower HbO was detected in the frontopolar cortex in the AIS group, and higher HbO was detected in the control group in the EC static balance state. In addition, contrast map analyses revealed that this activation shifted to the DLPFC region and showed a symmetrical distribution when the control and AIS groups were compared. This finding revealed that the postural control strategies of the individuals in the AIS group differed. The HbO reduction in FPC in the AIS group may characterize a deficit in shifting the focus from visual inputs to proprioception.

## 5. Conclusions

This study offers important insights into the neural mechanism by evaluating prefrontal cortex HbO activity in the AIS group under static balance EO and EC conditions. The AIS group had lower mean HbO values than the control group in the EC static balance condition, and its activity moved to the DLPFC region, showing more effective HbO activation from the frontopolar regions in the DLPFC and completely differentiating from the control group. This indicates that this group tended to employ conscious balance control by using the cortex more symmetrically and actively. In the EO condition in the AIS group, the maximum HbO value was reached in the frontopolar region, and this value was weakened and observed throughout the left frontopolar region, which may be an indication of a different compensation mechanism. The differences between the groups in both EO and EC conditions can be interpreted as a result of the adaptation mechanisms developed by the AIS group to compensate for postural imbalances.

Cortical findings indicate that scoliosis is not merely a mechanical disorder but also involves neurophysiological adaptations at the neural level, referring to changes within the central nervous system, particularly in brain regions responsible for postural control. Specifically, altered activation patterns in motor and sensorimotor cortical areas reflect functional reorganization processes, such as neural plasticity and modified sensory integration, which together characterize these neural-level adaptations.

Although the findings of this study are meaningful, the limited number of studies integrating fNIRS with postural stability assessment in the AIS population restricts direct comparisons with the existing literature. Given the lack of standardized protocols, our results represent an initial step toward understanding the neural basis of postural control. Future research should aim to strengthen the level of evidence by incorporating larger sample sizes, longitudinal designs, and multimodal neuroimaging approaches. Moreover, postural control studies using fNIRS in different clinical and healthy populations are also needed to establish comparative reference data and to explore population-specific neural mechanisms underlying balance. More detailed investigations are particularly required in terms of sensory–motor integration, postural control, and conscious movement strategies. Additionally, since the present study did not include recordings from other cortical regions, it is recommended that future studies explore whether similar patterns of compensatory neural activity are present in other brain areas, such as the temporal and parietal cortices, which may also play a role in postural regulation.

## Figures and Tables

**Table 1:** Demographic characteristics of the participants.

Demographic Characteristics				*p*-Value
Sex				0.439
Control Group	n	%		
Female	6	37.5%		
Male	10	62.5%		
AIS Group				
Female	7	46.67%		
Male	8	53.33%		
Age (years)	Mean ± SD	t	95% CI	0.08
Control Group	12.75 ± 2.64	−1.816	−3.65–0.217	
AIS Group	14.47 ± 2.615			
BMI (kg/m^2^)	Mean ± SD	Median (min–max)		0.782
Control Group	20.39 ± 5.31	19.24 (16.82–23.22)		
AIS Group	20.22 ± 3.60	19.14 (17.67–24.03)		
	Min	Max		Mean ± SD
Cobb Thoracic	5.0	27.8		13.8 ± 6.765
Cobb Lumbar	12.0	33.6		20.673 ± 7.009

Abbreviations: SD—standard deviation, CI—confidence interval, BMI—body mass index.

**Table 2:** Control–AIS group comparison in static balance eyes-open condition.

Channel	Control Group Mean ± SD	AIS Group Mean ± SD	*t*	Cohen’s d	*p*-Value
1	0.158 ± 5.545	0.888 ± 4.043	−0.417	−0.150	0.680
2	−1.426 ± 5.257	0.286 ± 3.563	−1.054	−0.379	0.300
3	0.409 ± 4.013	0.704 ± 8.179	−0.129	−0.046	0.333
4	2.086 ± 6.857	−1.064 ± 10.378	1.004	0.361	0.477
5	0.959 ± 7.093	1.471 ± 4.092	−0.244	−0.088	0.752
6	1.020 ± 6.165	1.135 ± 7.875	−0.134	−0.048	0.895
7	−0.769 ± 4.037	2.581 ± 6.373	−1.761	−0.633	0.206
8	−0.001 ± 3.417	1.938 ± 5.557	−1.179	−0.424	0.248
9	−1.539 ± 4.839	−0.053 ± 3.629	−0.962	−0.346	0.344
10	−0.554 ± 4.408	0.585 ± 4.789	−0.690	−0.248	0.527
11	1.081 ± 8.393	2.413 ± 4.074	−0.556	−0.200	0.268
12	−1.515 ± 3.993	2.679 ± 3.992	−2.920	−1.049	0.007 *
13	−1.618 ± 4.286	1.654 ± 3.345	−2.268	−0.848	0.030 *
14	−1.991 ± 3.503	0.820 ± 5.906	−1.625	−0.569	0.115
15	−0.696 ± 5.801	1.633 ± 4.975	−1.196	−0.430	0.241
16	−0.548 ± 3.533	−0.395 ± 6.101	−0.086	−0.031	0.932
17	−1.550 ± 5.068	2.190 ± 5.676	−1.938	−0.697	0.062
18	−1.702 ± 6.525	4.068 ± 7.267	−2.328	−0.840	0.018 *
19	−0.265 ± 4.995	0.602 ± 7.220	−0.391	−0.141	0.699
20	−0.260 ± 4.881	0.411 ± 7.374	−0.301	−0.108	0.766
21	−2.698 ± 12.304	−0.251 ± 6.874	−0.677	−0.243	0.664
22	−0.945 ± 6.158	0.898 ± 5.804	−0.856	−0.308	0.399

Independent-sample *t*-test, * *p* < 0.05.

**Table 3:** Control–AIS group comparison in static balance eyes-closed condition.

Channel	Control Group Mean ± SD	AIS Group Mean ± SD	*t*	Cohen’s d	*p*-Value
1	2.491 ± 4.063	−0.103 ± 6.122	1.367	0.510	0.183
2	2.306 ± 6.122	0.189 ± 5.211	0.976	0.365	0.338
3	1.841 ± 3.588	3.521 ± 8.358	−0.728	−0.272	0.895
4	3.556 ± 4.860	3.364 ± 12.283	0.057	0.021	0.405
5	1.521 ± 3.152	2.098 ± 4.4956	−0.381	−0.142	0.861
6	3.745 ± 5.115	2.150 ± 7.125	0.701	0.262	0.489
7	3.189 ± 6.343	0.526 ± 6.028	1.149	0.429	0.313
8	1.608 ± 5.133	2.459 ± 8.403	−0.336	−0.125	0.739
9	3.273 ± 6.255	−0.300 ± 6.772	1.475	0.551	0.152
10	2.548 ± 4.970	0.823 ± 5.042	0.924	0.345	0.293
11	3.238 ± 5.443	2.791 ± 9.237	0.162	0.061	0.599
12	3.243 ± 4.701	0.032 ± 5.646	1.672	0.624	0.106
13	3.343 ± 7.146	−1.075 ± 5.459	1.835	0.685	0.054
14	3.122 ± 5.738	−1.279 ± 4.230	2.301	0.859	0.029 *
15	5.105 ± 5.854	−1.483 ± 7.381	2.683	1.002	0.012 *
16	4.769 ± 5.232	−1.938 ± 7.421	2.852	1.065	0.008 *
17	2.101 ± 5.152	−1.693 ± 6.717	1.723	0.643	0.096
18	4.169 ± 5.806	−0.309 ± 7.666	1.791	0.669	0.048 *
19	2.519 ± 5.566	−1.223 ± 6.223	1.709	0.638	0.099
20	2.711 ± 5.752	5.752 ± −3.215	2.667	0.996	0.013 *
21	4.536 ± 8.394	0.042 ± 7.232	1.524	0.569	0.096
22	2.826 ± 5.971	−0.705 ± 6.720	1.498	0.559	0.146

Independent-sample *t*-test, * *p* < 0.05.

**Table 4:** Static balance: 3 different perspectives of control–AIS group topographic maps in eyes-open and eyes-closed conditions with topographic map color index (Min: −15 μmol/L; Max: 10 μmol/L). Visualization was performed using the most significant *t*-value change range for each group. Red and blue represent hyperactivation and hypoactivation, respectively.

Control		AIS
EO	** 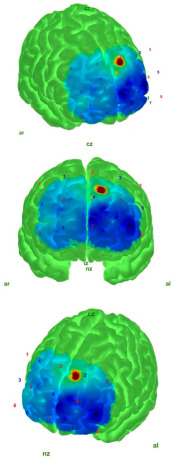 **	** 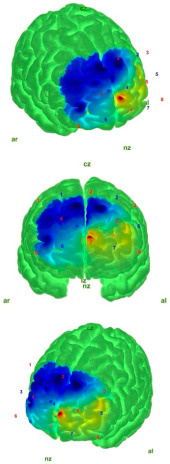 **
EC	** 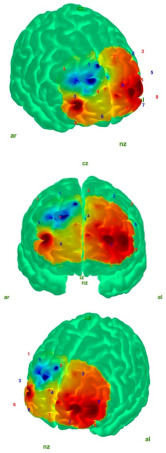 **	** 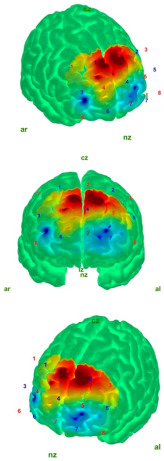 **

Abbreviations: AIS—adolescent idiopathic scoliosis (AIS); EO—eyes open; EC—eyes closed; nz—nasion; ız—inion; ar—right auricular; al—left auricular.

## Data Availability

The data presented in this study are available on request from the corresponding author. The data are not publicly available due to specific ethical and privacy considerations.
